# Phosphatized adductor muscle remains in a Cenomanian limid bivalve from Villers-sur-Mer (France)

**DOI:** 10.1186/s13358-022-00252-4

**Published:** 2022-06-03

**Authors:** Christian Klug, Liane Hüne, Rosemarie Roth, Michael Hautmann

**Affiliations:** 1 Paläontologisches Institut und Museum, Universität Zürich, Karl-Schmid-Strasse 4, 8006 Zurich, Switzerland; 2 Berlin, Germany

**Keywords:** Bivalvia, Soft-tissue preservation, Cretaceous, Palaeoecology

## Abstract

Soft-tissue preservation in molluscs is generally rare, particularly in bivalves and gastropods. Here, we report a three-dimensionally preserved specimen of the limid *Acesta clypeiformis* from the Cenomanian of France that shows preservation of organic structures of the adductor muscles. Examination under UV-light revealed likely phosphatisation of organic remains, which was corroborated by EDX-analyses. We suggest that the parts of the adductor muscles that are very close to the attachment are particularly resistant to decay and thus may be preserved even under taphonomic conditions usually not favouring soft-tissue fossilisation.

## Introduction

Bivalves have a long evolutionary history that dates back to the Early Cambrian (Geyer & Streng, 1998). Their diversity, abundance and metabolic activity steadily increased (Payne et al., 2014) and they have been a dominant taxon of benthic marine ecosystems since the Triassic (Friesenbichler et al., 2021). The autecology of extinct bivalve taxa is usually well-understood because of a close correlation between shell morphology and mode of life. However, preservation of soft parts is rare (e.g., Klug et al., 2005). Here, we report the preservation of soft tissue from the adductor muscle of the limid bivalve *Acesta clypeiformis* from the Cenomanian of France.

The adductores are usually differentiated in two parts that correspond to two different functions: (1) quickly contracting to close the valves under threat (quick muscle; fast, strong and short) and (2) keeping the valves closed firmly (catch muscle; slow, continuous, e.g., Bowden, 1958; Millman, 1967; Chantler, 2006; Simone, 2019; Eggermont et al., 2020; Castro-Claros et al., 2021). Accordingly, these muscle fibres can be differentiated functionally and morphologically into striated muscular fibres and smooth fibres. Their attachment to the shells is usually very strong (Castro-Claros et al., 2021), reflecting their importance in protecting the animal. Because of the required strength of this connection, the basal part of the adductors might become fossilized more easily than other soft tissues.

Fossilisation of mollusc soft parts is rare. Soft tissue-preservation is reasonably common in cephalopods (e.g., Kear et al., 1995; Klug et al., 2021a; Klug et al., 2021b; Hoffmann et al., 2021), it is very rare in gastropods (Sutton et al., 2006) and quite rare in bivalves (a list of references is given in Table 1).

**Table 1:** List of occurrences of fossilized soft tissues in bivalves (modified after Klug et al., 2005)

Age	Lithostratigraphy	Locality	Taxon	Soft-tissue	References
Middle Triassic (Anisian, Ladinian)	Muschelkalk	Baden-Württemberg, Germany	*Myophoria, Neoschizodus*	Mantle + adductor musculature, in- and excurrent siphons, blood vessels	Klug et al., 2005
Early Jurassic (early Pliensbachian)		Between Chipping Campden and Mickleton, Gloucestershire, UK	*Nuculana *(*Dacryomya*)* gaveyi*	Internal mould of intestine	Gavey, 1853; Cox, 1960
Middle Jurassic (Bajocian)	Wedelsandstein Fm	Aubach near Aselfingen, Germany	*Pholadomya fidicula*	Siphon	Mehl & Rehfeld-Kiefer, 1992
Late Jurassic (middle Oxfordian)	Upper Oxford Clay	Boarstall/Oxfordshire, New Farm/Oakley, England	*Gryphaea (Bilobissa) dilatata*	Adductores	Harper & Todd (1996)
Late Jurassic (Oxfordian)	Kimmeridge Clay Fm	South Ferriby, Lincolnshire, England	Bivalvia	Byssus	Todd and Palmer, 2002
Late Jurassic (Portlandian/Tithonian)	Portland beds	Wiltshire, Dorsetshire, England	*Laevitrigonia gibbosa*	In- and excurrent siphons, adductors, pallial ridge, gills/demibranchs, musculature, intestine	De la Beche, 1848, 1849; Mantell, 1843; Whyte et al., 1984; Spamer & Bogan, 1989; Whyte, 1992; Wilby & Whyte, 1995; Torrens et al., 2000
Late Cretaceous (early Cenomanian)		Villers-sur-Mer, France	*Acesta clypeiformis*	Bases of the posterior adductor muscle	This paper
Late Cretaceous (Coniacian to Campanian)	Niobrara Chalk, Smoky Hill Chalk Mb	Kansas, USA	Bivalvia	Musculature	Stewart, 1990
Late Cretaceous (Turonian, Santonian)	Grupo Bauru	São Paulo, Brazil	Bivalvia	Labial palps, demibranchs, musculature	Lopes de Simone & Mezzalira, 1993
Oligocene/Miocene	Pysht Fm	Merrick’s Bay, Washington State, USA	Xylophagaines and teredinid bivalves	Intestine, caecum	Kiel et al., 2012
Pleistocene		Oga Peninsula, Akita Prefecture, Japan	*Glycymeris yessoensis*	Internal mould of intestine	Chiba et al., 2014

Here we report soft tissue remains of a Late Cretaceous bivalve from France assigned to *Acesta clypeiformis*. We include analyses of its chemical composition and the significance of this discovery.

## Materials and methods

**Fig. 1:**
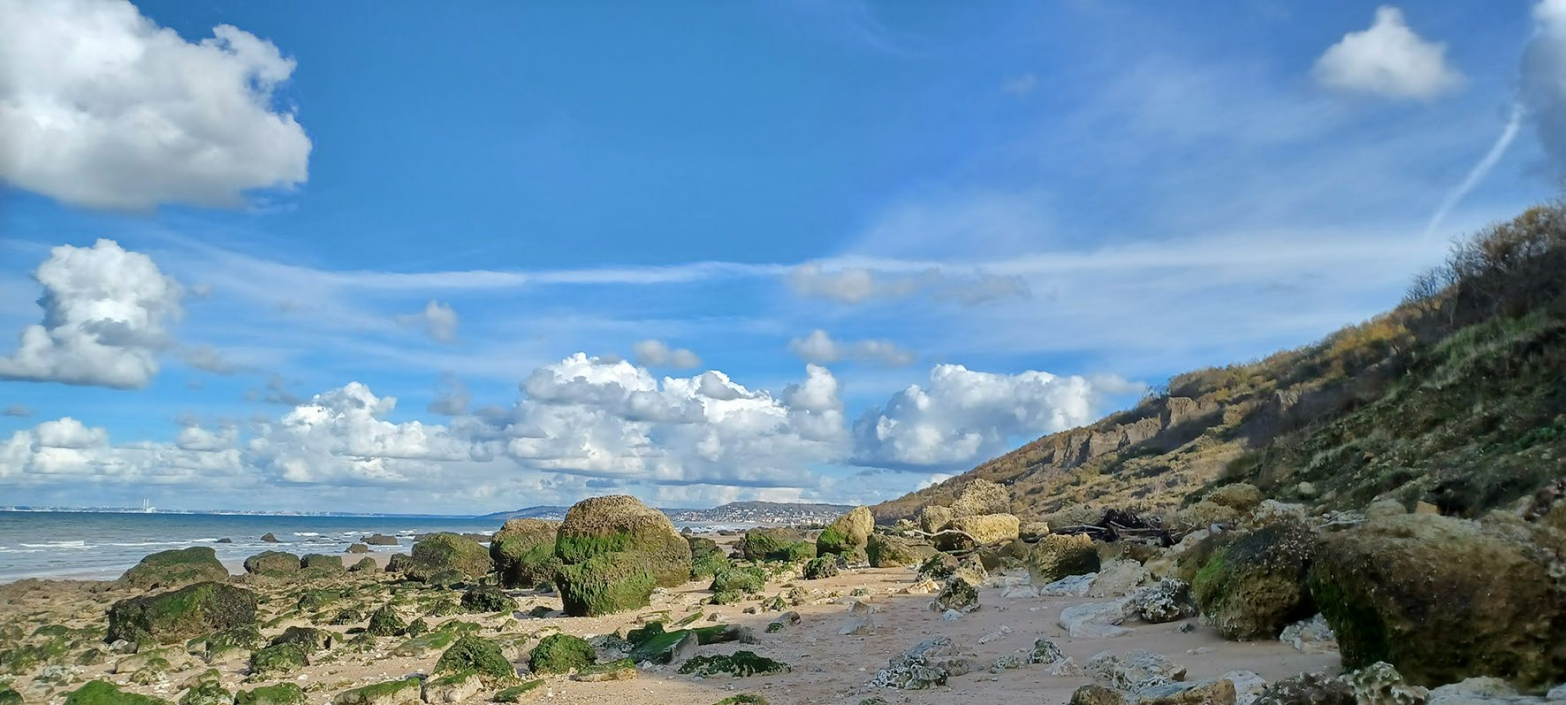
Falaises des Vaches Noires, France, with the early Cenomanian blocks on the beach, where the here described *Acesta clypeiformis *(d’Orbigny, 1847) was found (photo by LH)

Only one specimen of the limid bivalve *Acesta clypeiformis* with preserved soft-tissue is available. The specimen was found by L.H. at the Falaises des Vaches Noires. These cliffs are 110 m high and extend over 5 km along the coast of the Calvados Department (Normandy, France), between Villers-sur-Mer and Houlgate. Research on the Falaises des Vaches Noires began in 1776 with the work of the monk Jean-Francois Dicquemare (1775). Since then, it became a classic fossil locality of palaeontology (e.g., Brignon, 2015, 2017). The sediments composing these cliffs range in age from the late Callovian (Jurassic) to the Late Oxfordian. Oxfordian sediments crop out on the natural cut between the lowest level of the beach and the top of the lower cliff (Dugué et al., 1998). Set back further and more difficult to access, because of intense erosion, the early Cenomanian chalk (occasionally also late Aptian to late Albian) occurs at a height of 30–40 m above the beach (Beaugrand, 1884). Periodically, natural landslides are caused by groundwater emerging from the Cenomanian sediments above the Oxfordian marls (Costa et al., 2006; Duperret et al., 2005). These exposed parts of the section deliver scree, which slides down the slopes and ravines, sometimes all the way to the beach. The name “Vaches Noires” derives from large Cenomanian blocks that fell on the beach and were subsequently covered by algae and shells, reminiscent of a herd of cows (Fig. 1). The specimen of *Acesta clypeiformis* with fossilized soft-tissue remains was collected by L.H. from one of these blocks of glauconitic chalk from the Cenomanian. It is now stored with the number PIMUZ 37855 at the Paläontologisches Institut und Museum of the Universiät Zürich.

The remains of the right adductor muscle were examined by Andres Käch (Zürich) using a JEOL JSM-6010 tungsten cathode SEM with EDX v. 3.01, using a spot size of 50 μm without and with a high vacuum.

R.R. took photos of the bivalve under white artificial light and a UVA-handlamp (Hönle UV technology) with a Nikon D3X with a Nikon AF-S Micro Nikkor 105 mm 1:2.8 objective and a UV (UV-Filter MC Lotus from Kaiser Fototechnik) and polarising filters (Nikon Circular Polarizing Filter II). The colour of the UV-photos was corrected in Adobe PhotoShop 2021.

## Results

### Description

**Fig. 2:**
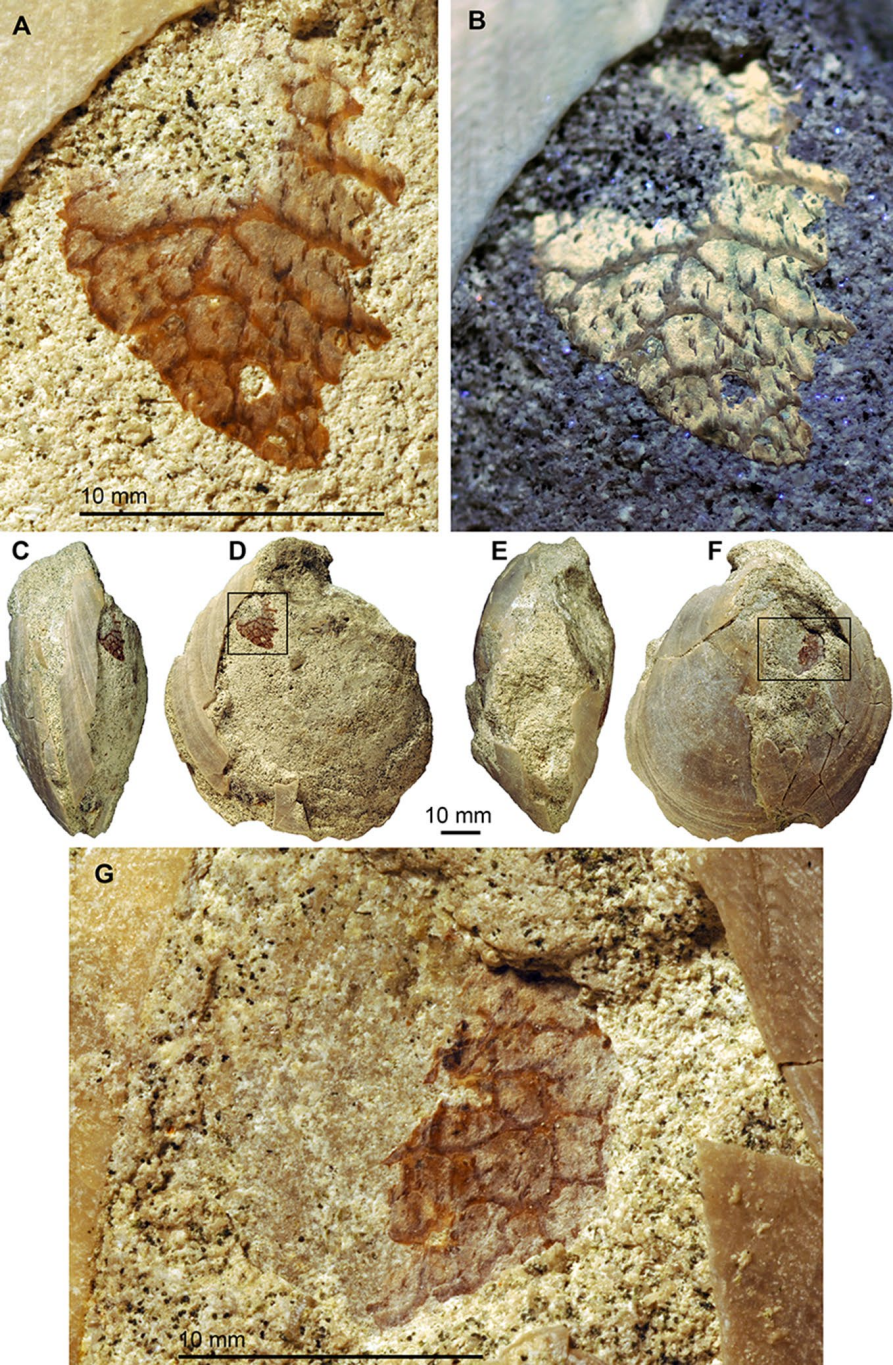
*Acesta clypeiformis *(d’Orbigny, 1847) with adductor muscle scars retaining phosphatized muscle remains. Cenomanian, Falaises des Vaches Noires, France. B taken under UV-light, **A**, **B** adductor muscle of the right valve (marked with a rectangle in D). **C**–**F** Photos of the entire specimen. **C** Anterior; **D** right; **E** posterior; **F** left view. **G** Adductor muscle of the left valve (marked with a rectangle in **F**)

Specimen PIMUZ 37855 (Fig. 2) is slightly deformed and is preserved with the external (calcitic) shell layer of both valves, which flaked off over a larger part of the right valve and, to a lesser extent, also over parts of the left valve. The internal filling consists of glauconitic marl. The specimen has been identified as *Acesta clypeiformis* (d’Orbigny, 1847), a widespread species of the family Limidae, figured, e.g., by Woods (1904, p. 26–27, Fig. 5, who included the species in the genus *Lima*). The articulated valves are 82 mm high, 73 mm long and 34 mm wide, although the latter value is altered by compaction. The shell of both valves flaked off around the attachment of the adductor, thereby revealing phosphatized remains of their bases, which is better visible on the right valve. There, the brown and beige remains cover an irregular triangular surface measuring 13.1 mm in length and 11.5 mm in height. The brown and beige surface shows a reticulate pattern of polygonal sectors between 1.5 and 2.5 mm wide, surrounded by brown lines of 0.3–0.4 mm width. These lines become lighter in colour postero-ventrally and show a faint striation with the same orientation (running postero-ventrally). The lines are referred to as brown parts, while the inside of these polygonal fields are referred to as beige parts. Within the beige parts, comma-shaped patches are distributed irregularly. They are about 0.5 mm long and between 0.1 and 0.2 mm wide. The reticulate pattern corresponds to the bundles of muscle fibres that made up the muscle.

### Chemical analyses of the adductor muscle remains

**Fig. 3:**
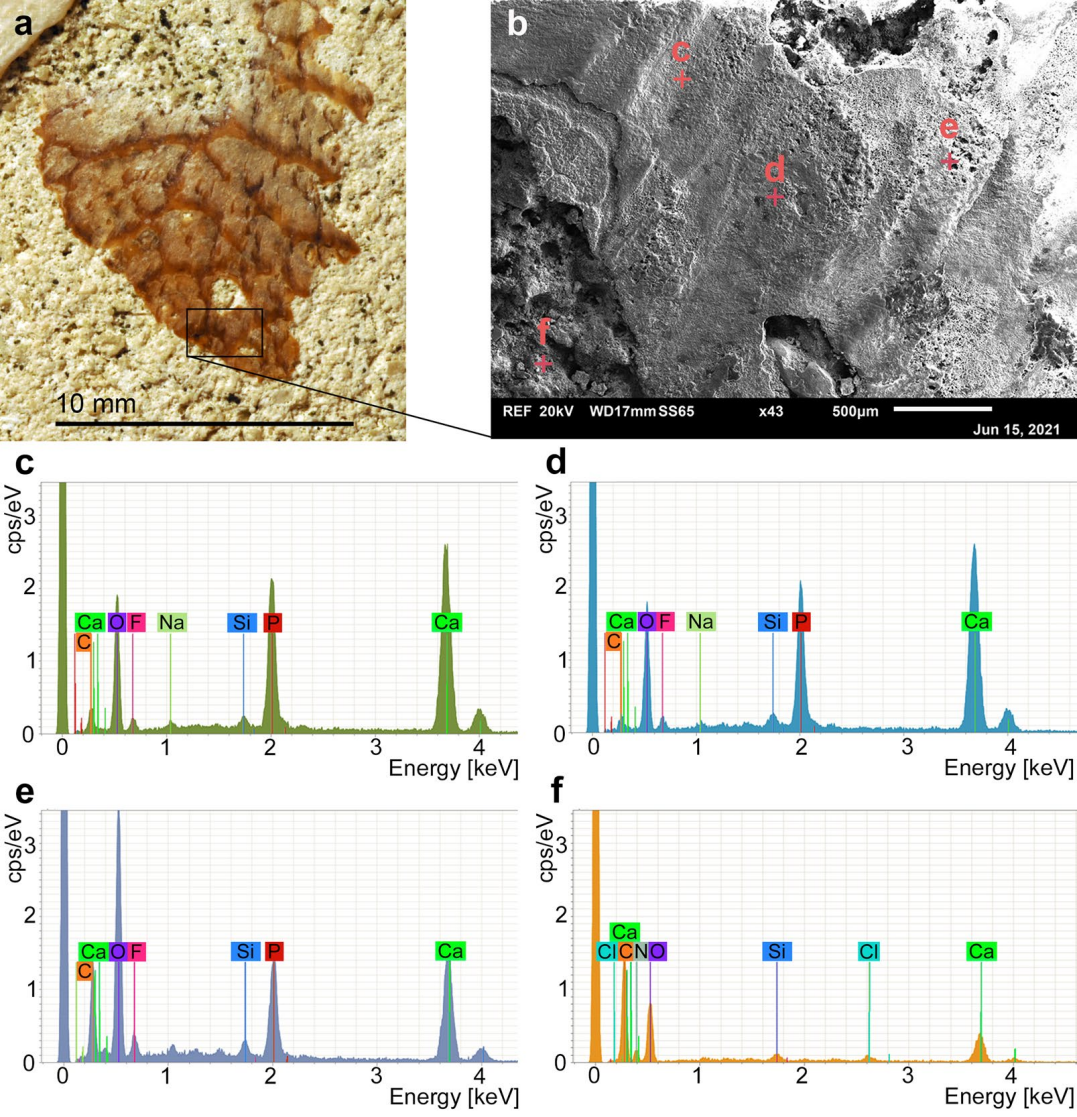
Element analyses of the adductor remains and the sedimentary filling of the bivalve shown in Fig. 2. For the weight percent values, see Table 2. **a** Detail of the bivalve showing the part that was measured (black rectangle). **b** Magnified detail taken by SEM, showing the spots, where the EDX-measurements were taken. **c** and **d** EDX measurements from the beige parts. **e** EDX measurement from the brown parts. **f** EDX measurement from the internal mould (glauconitic marl)

We ran several EDX analyses, four of which are presented here (Fig. 3, Table 2.). Three of the analyses were made in the brown parts and two of these three were made in the beige parts. The latter two analyses revealed similar amounts of oxygen (42–43 wt.%), calcium (32–35 wt.%), phosphorous (12–13 wt.%), carbon (4–7 wt.%) and fluorine (close to 4 wt.%). The analysis from the brown part revealed higher percentages of oxygen and carbon, but relatively less calcium and phosphorous. The analysis from the sedimentary matrix of the internal mould indicates a high content of calcium, carbon and oxygen, corroborating a CaCO_3_ composition.

**Table 2:** Results of EDX-analyses in weight per cent carried out on and next to the remains of the right posterior adductor muscle of *Acesta clypeiformis* (PIMUZ 37855)

Analysis	Where?	Mineral	O	Ca	P	C	F	Si	Na	N	Cl
Figure 3c	Scar, beige	Apatite/francolite	42.91	34.46	12.83	4.28	3.82	0.96	0.57	–	–
Figure 3d	Scar, beige	Apatite/francolite	42.47	32.37	12.45	6.56	3.67	0.74	0.66	–	–
Figure 3e	Scar, brown	Apatite/francolite	50.04	15.74	7.26	18.58	6.47	0.89	0.73	–	–
Figure 3f	Internal mould	Calcite	39.63	9.31	–	34.95	–	0.96	–	13.55	0.96

See Fig. 3 for the placement of the analyses

## Discussion

### What is preserved?

Limids are monomyarian (Cox & Hertlein, 1969); their single adductor is homologous with the posterior adductor of dimyarian pteriomorphs. On both valves, only the posteroventral parts of the proximal part of the adductor muscle is preserved. We assume that not the entire muscle is preserved but only the part very close to the shell attachment.

### How can its preservation be explained?

As demonstrated by Castro-Claros et al. (2021), Ca^2+^-ions play an important role in the attachment of muscles in bivalves. This suggests that the richness in calcium carbonate made the part of the muscle that was closest to the shell more resistant to decay and thus increased the likelihood of becoming phosphatized.

*Acesta* is a byssate limid bivalve. Modern representatives of this genus live in cold water or at great depths (Cox & Hertlein, 1969). Low temperatures would slow down disintegration of soft-tissues. However, Merles (2011) suggested that the water was rather warm during the Cenomanian in that region (see also O’Brien et al., 2017).

Phosphatisation of soft-tissues is known to occur in the sediment near the redox boundary (Allison, 1988a, 1988b; Briggs & Wilby, 1996; Briggs et al., 1993), often in combination with bacterial activity. In contrast to most other bivalves with fossilized soft-tissues (see Table 1), *Acesta* is not infaunal. Limids are usually attached to the substratum by the byssus, but they can release the byssus for swimming when attacked by a predator (Stanley, 1970).

Accordingly, we hypothesize rapid burial, which inhibited the decay of soft-tissues and brought the remains to the redox-boundary, thus enabling phosphatisation. It is also conceivable that local conditions within the closed shell were more important than the position of the redox boundary in the sediment and favoured phosphatisation. In addition, due to the proximity to the muscle insertion, we suggest that the resistance to decay of this part of the muscle is linked with the presence of raised levels of Ca^2+^-ions(Castro-Claros et al., 2021).

## Conclusions

We describe a Late Cretaceous fossil of a byssate epifaunal bivalve, which preserves those parts of the posterior adductor muscle that are the closest to the shell. We suggest that the combination of the proximal muscle with its abundant collagen fibres and the shell carbonate at the muscle insertion inhibited decomposition. Rapid burial likely stopped decay and enabled phosphatisation of these organic remains. This shows that under certain conditions, soft tissues may become fossilized in taphonomic contexts, where such preservation would normally not be expected.

## Data Availability

The single specimen is incorporated in the collections of the Palaeontological Institute and Museum of the University of Zurich (PIMUZ 37855).
